# How the informed relations between physical, digital and biological dimensions are changing the design practice, as well as the sustainability paradigm

**DOI:** 10.3389/fbioe.2023.1193353

**Published:** 2023-05-30

**Authors:** Carmen Rotondi

**Affiliations:** Department of Planning, Design, Technology of Architecture, Sapienza University of Rome, Rome, Italy

**Keywords:** bio-digital industry, material systems, material-based biological paradigms, growth-based paradigms, biological fabrication, biological qualities, human-nature-artifice cooperation, physical-digital-biological integration

## Abstract

In the “century of biotechnology”, a new form of “bio-digital industry” is emerging in which, thanks to increasingly sophisticated and digitized technologies that allow engineering and production on a biological quantum scale, it is possible to analyze and reproduce the generative, chemical, physical, and molecular processes underlying natural mechanisms. Inheriting methodologies and technologies from biological fabrication, bio-digital practices foster a new material-based biological paradigm that, bringing biomimicry to a material level, allows designers to observe substances and logic used by nature for assembling and structuring its materials, developing more sustainable and strategic ways for artifice manufacturing, as well as replicating complex, tailored, and emergent biological qualities. The paper aims to describe the new hybrid manufacturing techniques, demonstrating how the transition from form-based to material-based approaches also leads to the change of logic and conceptual frameworks in design practices, allowing greater alignment with the paradigms of biological growth. In particular, the focus is on informed relations between physical, digital, and biological dimensions, allowing interaction, development, and mutual empowerment between entities and disciplines belonging to them. Such a correlative strategy can help design to apply systemic thinking, from the scale of the material to that of the product and the process, paving the way to sustainable scenarios, not simply to reduce the human impact on the ecosystem but to enhance nature through original cooperation and integration forms between humans, biology, and machines.

## 1 Introduction

For a couple of years, we have joined the third decade of the “century of biotechnology”, so-called by Walter Isaacson (1999) in the journal Time. While in the 19th century, the mechanization of the loom gave birth to the First Industrial Revolution, and in the 20th century, the advent of integrated circuits on a silicon substrate ushered in the Information Revolution and restructured modern life, today, many predict that it will be the biotechnology the main driver of change (Ginsberg et al, 2017). In particular, it will be the combination of computational power–given by informatics and digital technologies–with a biological one–provided by biotechnologies, increasingly diffused, available, and economical (Carlson, 2011)—to upset the previous patterns and revolutionize the next future in healthcare and industry, economy and of course, design (Benjamin, 2011). Furthermore, contemporary biosciences are increasingly abandoning a predominantly descriptive character, transforming themselves into quantitative or engineering disciplines, developing tools (hardware and software), interests, and working ways that bring the scientific field closer to the design one as never before (De Lorenzo, 2014).

Therefore, new scenarios and opportunities for innovation open up to design, given by new technologies that allow engineering and production on a biological quantum scale and by new experimental domains of convergence with recent disciplines of biological derivation more similar from the point of view of logic and languages deriving from engineering and computation, longtime typical of design. Indeed, it is of no minor importance that, to date, the study of nature and its manipulation and reproducibility are dependent on computers and information technologies from which they also inherit languages and methodologies (Grushkin, 2020). These opportunities translate into new hybrid embodiments, which are placed in the interspace between the synthetic and biological dimensions and which exploit the possibility of giving living characteristics and functionalities to objects, buildings, and cities to design a more sustainable future from an environmental and ecological, but also ethical, social and cultural point of view.

In particular, a new form of “bio-digital industry” is emerging in which, thanks to increasingly sophisticated, digitized, nano, and biosynthetic technologies, it becomes possible to analyze and reproduce the generative, chemical, physical, and molecular processes underlying natural mechanisms (Estèvez and Navarro, 2017). Attention shifts from products to processes; the artificial is increasingly biological thanks to digital fabrication and generative design, and the living is increasingly artificialized thanks to biotechnologies, while the results are new concretizations able to embody almost all degrees of freedom of a natural phenomenon and externalize biological characteristics such as self-organization, redundancy, self-generation, multifunctionality, interactivity, reactivity, adaptation, and flexibility, therefore suitable for the complex and changeable contemporary living. The present paper focuses on these nascent bio-digital manufacturing forms, demonstrating how they are stimulating new multidisciplinary research strategies thanks to appealing advantages such as diverse forms of expression or performativity and novel aesthetics, as well as the possibility of reimagining sustainable production’s paradigms (Antonelli, 2012); but also how they are changing the design practices and conceptual frameworks. Inheriting methodologies and technologies from biological fabrication–originated in the context of tissue engineering and defined as “the use of biological materials and mechanisms for construction […] that mimic biological growth mechanisms “(Liu et al, 2010)—bio-digital practices foster a new material-based biological paradigm that extends computational and biological principles to matter itself, which becomes intrinsically sensitive, active and programmable. This leads to conceiving products as “material systems”, no longer made of homogenous parts with distinct functions but in which material-product-performance are designed as a single entity through information, growth and adaptation to the context. Bringing biomimicry at a material level, biofabrication allows designers to observe substances and logics used by nature for assembling and structuring its materials, developing more sustainable and strategic ways for artifice manufacturing, as well as replicating complex, tailored, and emergent qualities. In describing how the biofabrication paradigms are taken up by design, extending their application beyond the original fields of tissue engineering and regenerative medicine, we will focus on the informed relationships between the physical, digital, and biological spheres, possible thanks to sophisticated tools and technologies able to break down reality into its basic units–atoms, bits, genes–and translate datasets from one dimension to another, allowing interaction, development and mutual growth between entities and disciplines belonging to them. Such a correlative strategy can help design to apply systemic thinking, from the scale of the material to that of the product and the process, also leading to a new way of beholding nature, passing from its exploitation as a simple resource–a legacy of the previous Industrial Revolutions–to its conception as an organism with which to dialogue and experiment collaborative ways of transformation and improvement (Oxman, 2016; Natalio, 2018; Ramsgaard Thomsen and Tamke, 2019).

## 2 The legacy of biological fabrication: new material-based biological paradigms

Biofabrication is usually defined as the production of complex living and non-living biological products from raw materials such as living cells, molecules, extracellular matrices, and biomaterials (Mironov et al, 2009). The prefix “bio” implies that either raw materials, processes, or final products (or all of these) must be biology-inspired or biology-based; the term “fabrication” means making or constructing something from raw or semifinished material, as well as creating something different from its components (Mironov et al, 2009). In the fields of tissue engineering and regenerative medicine, it refers to the possibility of generating complex constructs that mimic the complexity and the heterogeneity of biological tissues and organs through top-down (bioprinting) and bottom-up (bioassembly) processes in order to support and guide cell growth and regenerate the tissue of interest, as well as create biological models *in vitro* (Moroni et al, 2018). For example, with 3D bioprinting is nowadays possible to create scaffolds, two-dimensional or three-dimensional and porous structures, preferably biodegradable and which can mechanically support cell growth. They are modeled in CAD software to reach specific shapes, mechanical properties and porous distribution, and then they are printed with different layer-by-layer techniques. The choice of scaffold material is also essential in bioprinting, which must have specific characteristics of processability, surface (hydrophilicity and roughness), biodegradability, and biocompatibility, and in this case, nanotechnologies can be of great help allowing to equip the material of specific biochemical and biophysical nano-characteristics to direct cellular behavior (Di Marzio et al, 2020).

In design practice, biofabrication technologies and methodologies are applied to experimental structures, architectures, and consumer products, ushering us into a new material age driven by technologies and processes increasingly similar to nature. In fact, the biological fabrication paradigm does not refer simply to manufacturing with biological or living building blocks, but that design and fabrication procedures are aligned to the natural ones of growth and to the informational processes at their base to produce complex structures that mimic the intelligence, the specificity and the active qualities of “naturally developed” entities. Contrary to a form-based approach to design, in nature, the typical hierarchical sequence of form, structure, and material design is typically bottom-up reversed, as the material informs the structure, which informs the form (Vincent, 1982). Materials are designed for highly specialized functions rather than being assigned preconceived shapes. New technological means allow the combining biological and digital computation so that the control and structuring of the material organization are informed by environmental and material performance constraints, establishing seamless relationships between material-product-performance. Moreover, a growth-based approach that starts from the material structuring accordingly to biological paradigms and information datasets allows the transfer of many properties such as structural heterogeneity for specific adaptations and performativity (Oxman, 2011); responsiveness; dynamic and transformative behaviors (sensing, motion, shape change); bioreceptivity, and so on.

From this perspective, we distinguish within the material design practices at the intersection with biology a specific pathway that we can define as “bio-digital fabrication” or “digital biofabrication,” which is different from other practices based on the use of living matter. In particular, we can distinguish four macro-fields (Collet, 2013; Camere and Karana, 2017) that also correspond to a more general difference of biofabrication from other living matter-based technological platforms (Mironov et al, 2009): i) “growing design,” that matches with biomanufacturing and refers to the possibility to produce sustainable materials and biomolecules harnessing living matter as a raw material through making, crafting and tangible practices; ii) “augmented biology,” that matches synthetic biology and refers to the synthesis of new biological entities and materials from genome engineering; iii) “bio-digital fabrication,” that complies with biofabrication and stretches the possibilities of biological manipulation adopting computational tools, digital advanced manufacturing technologies and mathematical modeling tools involving nature as object of design processes; iv) “biodesign fiction,” which does not properly focus on the productive paradigms of the present, but imagines–often with conceptual visions–far provocative futures for advanced biotechnological developments.

### 2.1 Physical and digital: first steps towards growth-based paradigms

The first aspect of bio-digital fabrication is a strong connection between digital and physical (material) spheres that results in aesthetically and functionally augmented materialities, in which living and intelligent qualities are given by the intrinsic properties of the matter itself (Tibbits, 2017). Besides objects equipped with artificial intelligence, materials themselves may be informed and rewritten by computational facilities (e.g., cognitive computing, next-generation computer visualization) and digital procedures (e.g., digital fabrication techniques, digital material representations, algorithmic form-generation methods), able to work at multiple scales (e.g., 3D microfabrication). These research paths are particularly relevant in biofabrication for tissue engineering and bioprinting to fabricate scaffolds with hierarchical, dynamic, and heterogeneous structural properties that mimic and support cell growth behavior. In design, it allows products and architectures with optimized biological properties starting from the physicochemical characteristics of the material rather than the integration of electronic components or specific support structures, with consequent advantages also for sustainability. Moreover, thanks to the democratization of digital manufacturing and open source paradigms–as the numerous implementation attempts of DIY bioprinters (Bharadwaj and Verma, 2021) –, contemporary advanced manufacturing processes increasingly include biopolymers (e.g., gelatin, pectin, chitin, chitosan, collagen, alginate, cellulose) allowing the design to experiment with bio-based materials and harness them as a vehicle for intelligent behaviors.

**FIGURE 1:**
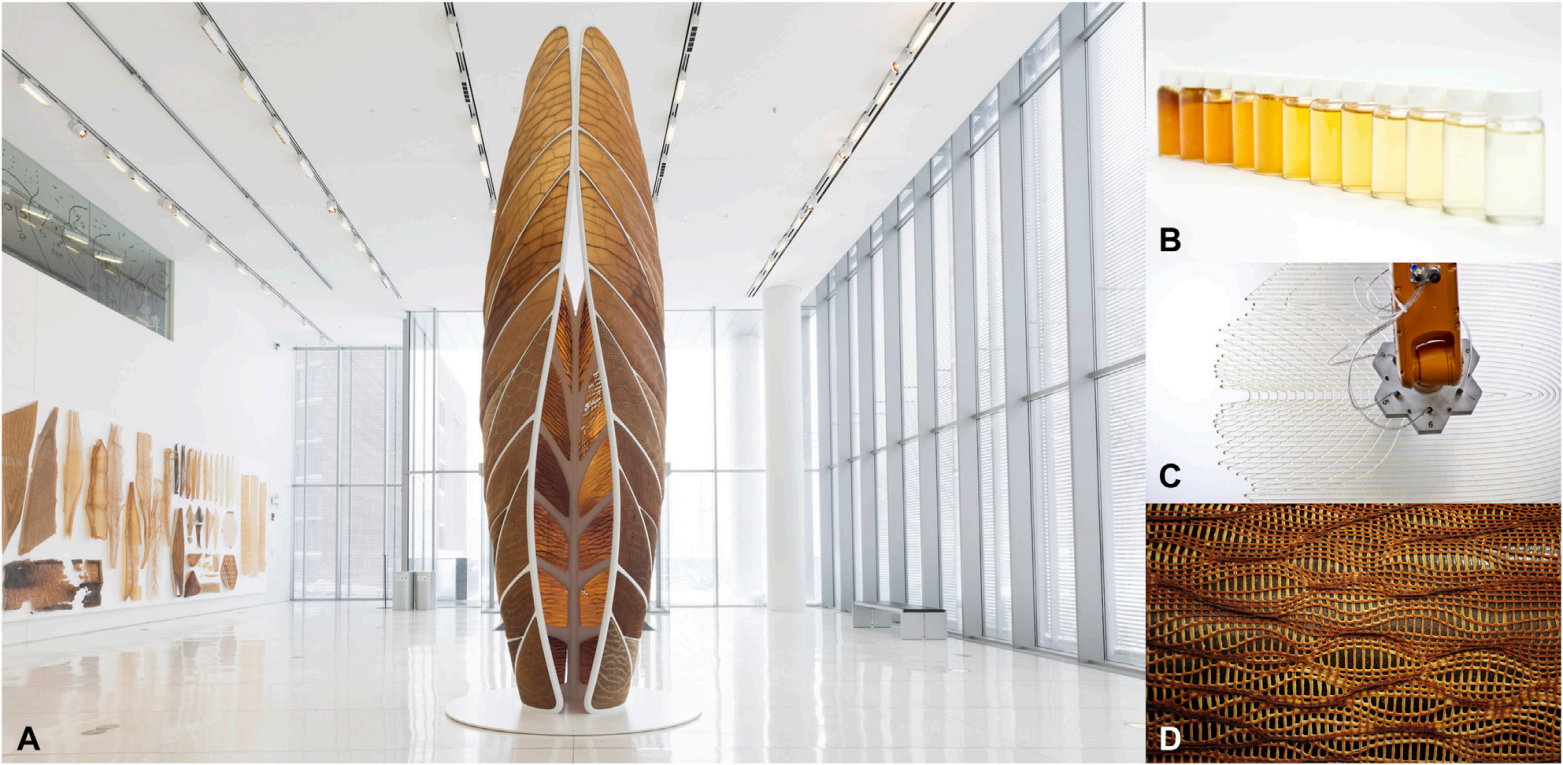
“Aguahoja” project by Neri Oxman and The Mediated Matter Group at MIT. It results in a pavilion made of 3D-printed biopolymers **(A)**. Different biopolymer concentrations in water give different optical and mechanical properties **(B)**. These composites are 3D printed with a robotic arm and combined **(C)**, resulting in a functionally graded skin **(D)**. Copyright: Neri Oxman (all the images are retrieved from https://oxman.com).

An example is the “Aguahoja” project by Neri Oxman and The Mediated Matter Group at MIT (Mogas-Soldevila, Duro-Royo and Oxman, 2014; Mogas-Soldevila and Oxman, 2015). It is a Water-Based Digital Fabrication platform that converts abundant biopolymers (cellulose, chitosan, pectin) into high-performance, sustainable materials which become printable once mixed with water. On a physical level, they analyzed multi-scale mechanical properties and different degradation rates of biopolymers, also depending on the water quantity (Figure 1B). On a digital level, they experimented with a collection of hardware, software, and wetware tools and technologies to alter and control the properties of the substances with computational patterns and structures, like transparency, strength, and decomposition modality (Figure 1C, D). The final result was a pavilion, entirely 3D printed and biodegradable, from water to water as in nature (Figure 1A).

Other examples are the numerous experiments which, through the use of parametric design, digital manufacturing tools (CNC machines, multi-material 3D printing, robotic fabrication, digital weaving) and cellulose-based biomaterials, aim to equip material systems with programmed hygroscopic behaviors, able to change shape on humidity variation according to specific directional patterns. The Self Assembly Lab at MIT has experimented with multi-material printing of cellulose-based (moisture absorbing) and PLA (hydrophobic) filaments to create programmable wooden structures that bend along specific directions (Correa et al, 2015), also allowing 2D printed elements to self-assemble into 3D structures (Papadopoulou, Laucks and Tibbits, 2017). They are in the field of 4D printing, which consists of 3D-printed multi-material structures that change shape and physical property over time (Tibbits, 2014), and that is increasingly used also in biomedical fields to imitate the dynamic behavior of native extracellular matrices (Moroni et al, 2018). In fashion design, Jane Scott (2018) designs “Programmable Knitting”, shape-shifting moisture-responding anisotropic behavior of pulp-based fibers woven into textiles to determine the geometry related to each deformation and integrate it into the design process by using parametric design.

From these examples, we can deduce how design practice and product imagination are changing. A permanent confrontation of information and materialization takes place, where digital programming influences the material, and the specific parameters of the material influence the digital models (Johns, 2014). Designers can thus rely on a new form of digital mediation between “real” matter and “virtual” information, leading to new formal and functional impulses and contexts (Holzbach, 2014).

### 2.2 Physical, digital and biological: an hybrid act of building and growing

Developments in scientific disciplines such as molecular biology, genetics, or synthetic biology extend the properties of the digital world to nature itself, which becomes understandable to the last detail, programmable and manipulable. Moreover, the paradigms of biofabrication extend not only to bio-derived or biobased substances but also to living matter, which is included in bio-digital design projects as a vehicle for intelligent behavior and as an interactive interface (bio-sensors and bio-actuators) with the environment and with users. Conceptually, in fact, biofabrication processes can be divided into two broad categories; either the scaffold is fabricated independently before the cellular component is added, or the scaffold is fabricated currently with the cellular component (Burg and Burg, 2014), so in this last case, we can talk about “bioinks” containing living matter directly printed under controlled conditions (Hospodiuk et al, 2017). In both cases, designers are experimenting with new manufacturing processes that involve directly living matter (such as algae, fungi, and bacteria), trying to bring them even to larger scales than those generally used in the biomedical field, relying on greater consilience with the biosciences and increasingly sophisticated advanced manufacturing tools.

**FIGURE 2:**
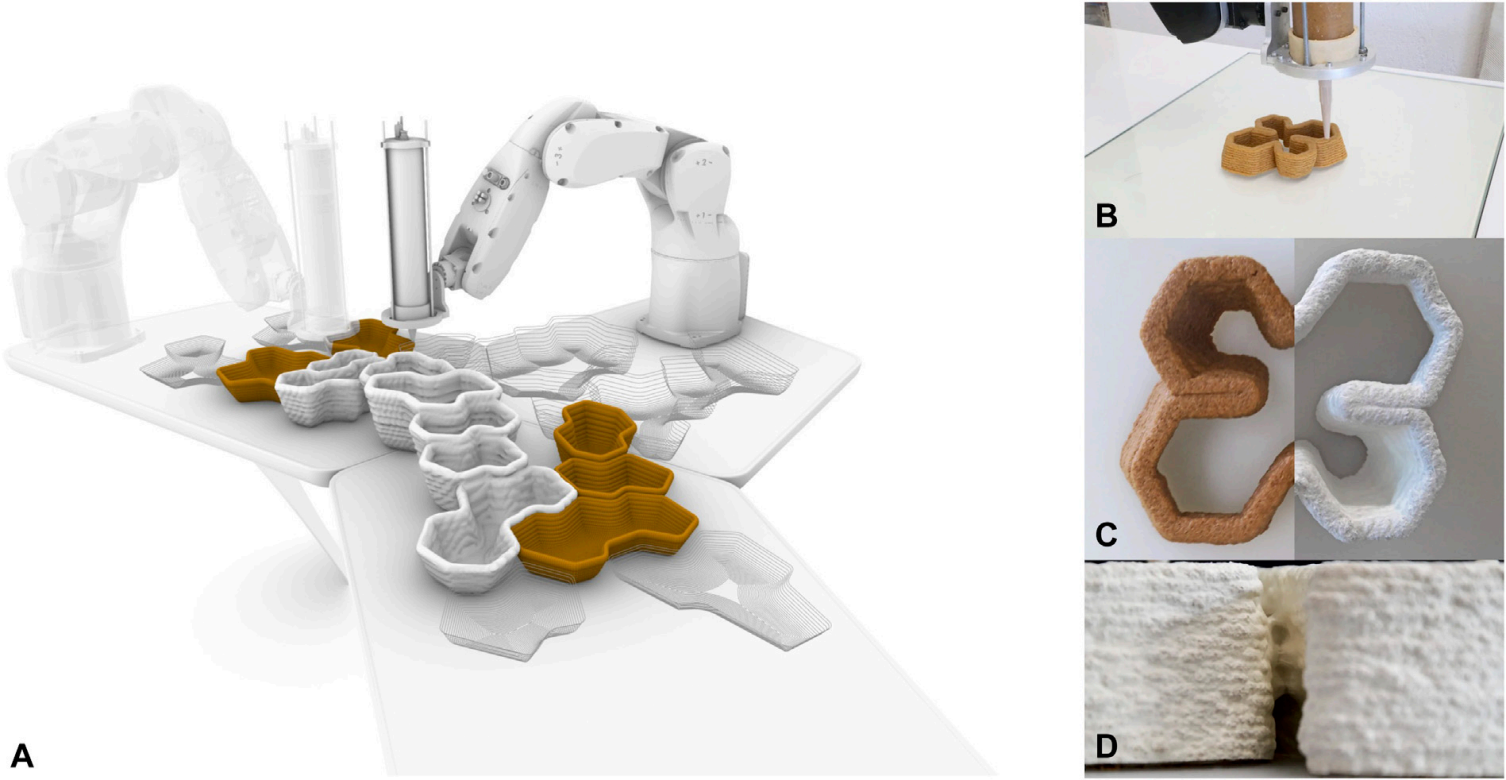
“Bio-ex-Machina” project by Officina Copruscoli and Co-de-it. It results in a set of customized, on-demand items of furniture 3-D printed with a robotic arm **(A)**. This last is customized for printing large-scale scaffolds made of organic waste and mycelium spores **(B)**. The mycelium grows and transforms the scaffolds into light and strong structures **(C)**. The ability of the mycelium to create filamentous links is also helpful for naturally developing joints between the pieces **(D)**. Copyright: Officina Corpuscoli and Co-de-it (all the images are retrieved from https://www.corpuscoli.com).

For example, professor Marcos Cruz and his Bartlett School of Architecture (United Kingdom) team have developed a bioprinting technique for large-scale, custom-printed immobilization of microalgae (Malik et al, 2019). They combine physical exploration of alginate-based hydrogel under printing conditions, digital exploration of algae’s growth pattern and printer’s parameters, and biological understanding of algae’s surviving abilities and absorbency capacity. Many innovative and sustainable scenarios arise from this research: they imagine a future in which algae ramifications cover buildings’ facades, insulate, guide rainwater, purify cities’ air, and establish new aesthetics. These experiments aim to facilitate biological growth on different supports and direct it according to specific needs. It is also the case of the “Silk Pavilion” realized by the Mediated Matter Group at MIT and characterized by a domed-shaped scaffold made by an intricate pattern of silk threads robotically fabricated and digitally designed to guide silkworms that filled all the gaps in the pattern with their silk, guided by the density variation predefined by the robotic-made threads and light variation (Oxman et al, 2014). In some cases, when the living matter is more processable and the required environmental conditions are less rigorous, designers have tried to propose forms of large-scale bioprinting directly involving living cells. For example, in the “Bio-ex-Machina” project, Maurizio Montalti and the Co-de-it team (2016) have printed with a customized robotic arm a mixture based on organic waste and mycelium spores to create new morphologically complex types of furniture, whose structural and aesthetic properties are given by the combination of algorithms, robotic behaviors and biological growth of the mycelium (Figure 2). Erik Klarenbeek experimented similarly in “Mycelium Chair” (Fairs, 2013). Another example is the project “Urban Reef,” developed by the Dutch designers Pierre Oskam and Max Latour, which involves complex 3D-printed geometries and porous materials, including ceramics and other composites made from coffee grounds and mycelium which, interacting with moisture in the environment, will be able to pass through and encourage the growth of various fungi (Marchese, 2021). Similarly, the University of Virginia researchers have invented a method of 3D printing with seed-impregnated soil, which could be used to create walls and roofs teeming with plant life (Walker, 2022).

So, the biological sphere comes into contact with the previous ones of digital and physical, bringing the material revolution to take a step forward and, consequently, the practice of design to explore further ways of innovation. In this case, it is not digital that gives the living qualities, but it is the matter itself that brings them into the material system, with consequent advantages from the point of view of environmental sustainability, but also with new aspects to understand and manage. On one side, this can be relevant to design more complex and long-lasting interactive materialities, exchanging information and feedback loops with other entities and the environment. On the other side, a vital component of unpredictability comes out and–besides the rise of ethical issues to which we have to answer–the programmability of these new entities will depend on our capability of establishing a synergistic relationship with them, given by an information exchange from us to the system and *vice versa*. Of course, digital facilities can help designers to prevent and control such complex processes, for example, through simulations or scaffold engineering. However, if so far computing and digital fabrication have translated our physical-tangible comprehension of the artifact from manual exploration to the ability to break it down into three space coordinates, now we move forward a fourth-time dimension that we should learn to manage, managing living matter.

## 3 Conclusion

The broad spectrum of potential applications and the rapid development of various biofabrication methods strongly suggests that biofabrication can become a dominant technological platform and a new paradigm for 21st-century manufacturing (Mironov et al, 2009). By bringing biomimicry to the material level and supporting the biological growth mechanisms, it stimulates the birth of new hybrid manufacturing techniques able to align objectives and interests of science and design, as well as meet the composite needs of society with sustainable, customizable, intelligent and specific application scenarios and product concepts. In particular, the integration of physical, digital, and biological processes and technologies stimulates the production of digitally manufactured objects and structures able to accommodate and grow biological organisms, perfectly combining the environmental needs of de-materialization and mono-materiality with the productive ones of flexibility and customization, as well as with the biological qualities and social needs of multifunctionality, autonomy, and interactivity. So, biofabrication paves the way to sustainable scenarios, not simply to reduce the human impact on the ecosystem but to enhance nature through original cooperation and integration between humans, biology, and machines.

This can be relevant also from an ethical point of view: new technological ability is, in fact, leading postdigital science, where biology as digital information, and digital information as biology, are now dialectically interconnected (Peters, Jandrić and Hayes, 2021). As Pasquinelli (2011) said, biodigitalism emerges as a new episteme concerned with the living—with bios—and the intersections between genetic and digital codes that continue to furnish the ‘new biology’. In other words, the evolving co-evolution of two overlapping systems (bios and techne) has accelerated interactions over the last couple of decades, weaving biodigital technologies into our lives as digital technologies have done. More than a technological change, this biodigital convergence may transform how we understand ourselves and cause us to redefine what we consider human or natural (Policy Horizons Canada, 2020). In this sense, biodigital experimentations can be relevant also to widen the bioethical field beyond the traditional objective of the ecological dimension as a set of resources useful for the survival of the human species (Ten Have, 2020)—building a bridge between the natural sciences and the human sciences –, towards continuous and philosophical reconfigurations of the whole system of life, including biohumanities, digital humanities, biopolitics, “bioepistemologies” (or evolutionary theories of epistemology), and evo-ontologies (Peters and Jandrić, 2019).

## Data Availability

The original contributions presented in the study are included in the article/Supplementary Material, further inquiries can be directed to the corresponding author.
